# Dimensions of Self-Reported Driving Difficulty in Autistic and Non-Autistic Adults and their Relationship with Autistic Traits

**DOI:** 10.1007/s10803-021-05420-y

**Published:** 2022-01-12

**Authors:** Elizabeth Sheppard, Editha van Loon, Danielle Ropar

**Affiliations:** 1 School of Psychology, University of Nottingham, University Park, Nottinghamshire, Nottingham, NG7 2RD UK; 2 Department of Psychology, Nottingham Trent University, Taylor Building, Burton Street, Nottingham, NG1 4BU UK

**Keywords:** Autism, Autistic traits, Driving, Driving difficulties

## Abstract

A survey asked autistic and non-autistic people about the driving difficulties they experience and their autistic traits. Principle components analysis was used to identify how reported difficulties clustered together in each group, and regression was used to determine which subscales of the Autism Spectrum Quotient predict these factors. For autistic drivers three factors of driving difficulty emerged: a Driving Executive factor, predicted by Attention Switching; a Driving Understanding factor, predicted by Communication; and a Driving Social Interaction factor, predicted by Attention Switching. For non-autistic drivers only one Driving General factor emerged, predicted by Communication. This suggests autistic people may experience at least three distinct domains of difficulty when driving which may relate to their particular profile of autistic features.

Driving is an important skill, which influences quality of life/wellbeing for autistic people^1^ through increased independence and reduced social isolation (Feeley et al., 2015). Driving can also facilitate better access to employment, opportunities for which have been found to be reduced for autistic people (Renty & Roeyers, 2006). Lack of available transportation is frequently cited as a barrier to accessing employment by autistic people (Feeley, 2010). Alternatives to driving such as public transportation may raise challenges for autistic people, such as issues with scheduling, overcrowding, and dealing with other passengers (Lubin & Feeley, 2016) as well as sensory challenges, further increasing the value of gaining a driving license. However, despite the advantages of attaining a driving license, estimates suggest that the proportion of autistic adults who do so is substantially lower than for non-autistic adults. In a study that followed over 50,000 individuals who reached licensing age in New Jersey, including around 600 with a diagnosis of autism, only around 33% of those with autism obtained their license while over 80% of non-autistic adolescents did over the same period (Curry et al., 2018).

A few previous studies have sought to identify possible causes of driving difficulty in autism using survey methods, although research in this area is still limited. For instance, Cox, Reeve, Cox and Cox et al. (2012) carried out an online survey of 123 parents or caregivers of autistic individuals where the median age of the autistic son or daughter was 19. The survey focused on parental concerns about their son or daughter driving. They reported that around 70% of parents felt that autism had negatively impacted their son or daughter’s driving and a similar proportion were worried about their child driving. Cox et al. also asked parents to rate the impact they felt that certain characteristics of autism had on their child’s driving, finding that multitasking, attention, and understanding non-verbal communication were perceived as the areas most likely to influence driving.

Autistic adults themselves have also been asked about their experiences in relation to driving (Daly et al., 2014), in a study of 78 autistic adult drivers and 94 comparison individuals. The Driver Behaviour Questionnaire (Reason et al., 1990) was used, which asks about 50 problematic driving behaviours falling into four categories: intentional violations (e.g. overtake on the inside because the other driver is going too slow); unintentional violations (e.g. accidentally speeding); mistakes (e.g. get in wrong lane when approaching junction); and slips/lapses (e.g. misread signs, turn on one thing when you intend to activate something else). Autistic adults reported more intentional violations, mistakes and slips/lapses, but not more unintentional violations. Autistic respondents also rated their own driving ability as lower, were more likely to place voluntary restrictions on their own driving (e.g. not driving at night) and were more likely to report being in an accident where they hit someone or something. A more recent survey included responses from a range of different informants including young autistic drivers, their parents/caregivers, and driving instructors, who were asked specifically asked about their experiences in relation to the process of learning to drive (Ross et al., 2018). This largely corroborated previous survey reports with multitasking, responding to unexpected events, and violating rules when needed being reported as key issues. Additionally, communication was reported to be a barrier to the learning process. While self-report questionnaires are open to the possibility of over or under-estimation of an individuals' abilities, requiring group differences to be treated with caution, confidence in these findings is increased by the broadly consistent views of different informant groups across studies. Moreover, self-perceptions of one’s ability, whether accurate or not, are important given that they might impact a person's willingness to drive or engage with the learning process.

Although autistic people have reported a wide range of difficulties with driving, little is known about whether and how these many disparate difficulties cluster together for autistic people, and whether this is different from how driving difficulties present in non-autistic populations. Moreover, it is currently not known to what extent particular driving difficulties map onto specific aspects of the autism phenotype. Autism is characterised by differences in social communication and a tendency to have restricted interests/repetitive behaviours (American Psychiatric Association & DSM-5 Task Force, 2013). For instance, difficulties with doing multiple things at once while driving might relate to the extent of difficulty an individual has in switching their attention or executive function (Hill, 2004; which relates to having restricted interests/repetitive behaviours), while difficulties predicting the behaviour of other road users might relate to the degree of social difficulty (difficulty with Theory of Mind; Baron-Cohen et al., 1985) an individual has. Equally, it is conceivable that some autistic traits might be associated with fewer difficulties in some domains. For instance, attention to detail (which has been related to weak central coherence; Frith, 1989/enhanced perceptual functioning in autism; Mottron & Burack, 2001) could potentially be associated with good ability to judge physical characteristics of the road environment such as distances between objects. Understanding how difficulties with specific aspects of driving relate to particular autistic traits or features is important as, given the heterogeneity of autism, this could give insights into which aspects of driving a particular autistic individual might find challenging, based on knowledge of their profile of autistic features. In turn, this can inform us whether multiple different programmes of intervention will be needed in order to individualise training to a person's particular pattern of strengths and difficulties.

Here we report a survey where we asked autistic and non-autistic adults about the extent to which they experienced a wide range of difficulties while driving. While previous studies focused only on those autistic individuals who had successfully obtained a license or were in the process of doing so, the study we report here also included autistic and non-autistic people who did not hold a license either because they did not complete driver training or because they have decided not to attempt to learn. Little is currently known about the views and experiences of driving in autistic non-drivers. Given the potential benefits of learning to drive, the experiences of this group seem particularly important to understand as interventions may be most needed for this group. Principle Components Analysis was used to determine whether driving difficulties cluster for each population, and whether these clusters may differ. In addition to this, all participants were asked to complete the Autism Spectrum Quotient (Baron-Cohen et al., 2001). For the autistic participants, this was used to give an indication of the level and type of autistic features they had, to be used as a predictor for reported driving difficulties. For non-autistic participants, this enabled us to explore the possibility that having higher levels of subclinical autistic traits would also be associated with greater difficulty when driving.

In summary, this study aimed to determine 1) the frequency and structure of perceived driving difficulties in autistic and non-autistic drivers; 2) whether specific autistic traits predict perceived driving difficulties in autistic and non-autistic drivers; 3) the frequency of perceived driving difficulties in autistic and non-autistic non-drivers; 4) whether specific autistic traits predict perceived driving difficulties in autistic and non-autistic non-drivers.

## Methods

### Participants

The survey was posted to a wide variety of websites and online forums including several autism forums, general participant recruitment forums, and Reddit. Cover information was provided to indicate that the study would focus on driving experiences and that participants could respond regardless of their driver and diagnostic status. The study was anonymous although participants were given an option to leave an email address if they wished to receive information about the findings of the survey. Participants were not compensated for their participation although they could choose to enter a prize draw to receive one of three Amazon vouchers after the survey had closed.

A total of 388 participants responded. Of these, 162 indicated that they had a current diagnosis of autism (Autism, Aspergers Syndrome or Autism Spectrum Disorder) while 215 indicated that they did not have any autism diagnosis. A further 11 participants stated that they suspected that they were autistic but had not received any formal diagnosis. Due to the uncertainty of the diagnostic status of those participants who reported suspecting that they were autistic, as well as the small number of participants concerned, those individuals were omitted from between-group analyses.

The majority of respondents reported living in either the UK or the US, although responses came from many different countries including Canada, Germany, the Netherlands, Ireland, Norway, Slovenia, Austria, Sweden, Australia, Denmark, Hungary, Italy, Jamaica, Mexico, France, Belgium, Greece, Malta, India and Singapore. Details of the autistic and non-autistic participant groups are shown in Table 1.

**Table 1:** Participant details

	Gender	Age/years	AQ score	Location	Education
Autistic	86 M, 76F	31.59 (11.34)	38.97 (6.74)	46% UK; 30% US; 24% other	31% school level; 18% higher level; 44% university; 7% other
Non-autistic	99 M, 112F, 4 other	28.48 (12.31)	20.95 (8.98)	33% UK; 48% US; 19% other	24% school level; 18% higher level; 42% university; 16% other

### Measures

A flexible survey was created where the questions differed depending on the participant’s particular driver status. The survey was developed using LimeSurvey software and presented via a dedicated website for driving-related research at the University of Nottingham. The questions included in the survey were derived from previous survey studies on driving difficulties in autism. The questions were reviewed (for both content and clarity) and revised based on feedback from two driving instructors, one of whom was autistic themself and had considerable experience teaching autistic learners.

The first section of the survey asked a number of demographic questions such as about the participant’s age, gender, country the participant lives in, education and employment status, as well as the participant’s diagnosis.

The next section of the survey asked details about the participants’ driver license status. The first of these asked whether the participant held a license and subsequent questions differed depending on how this question was answered. Participants who responded that they currently held a license were asked how long the license was held, how many times they took the test, how many lessons they required with a driving instructor before passing the test, how much practice they had before passing the test, how many miles they drive in a typical month, whether or not they own a car, and whether they had been in an accident over the past year as a driver. Participants who indicated they were currently learner drivers were asked how many lessons they had had, and how many times they had taken the test so far. Participants who indicated that they did not have a license and were not currently learning to drive were asked if they had tried to learn in the past but discontinued prior to obtaining a license.

In the following section, participants who responded that they currently held a driving license or were learning to drive were asked to rate how frequently they experienced various difficulties when driving on a five-point scale where 0 points indicated they never experience the difficulty while 4 points indicated they experience the difficulty very often. These included: Difficulties with physically operating the vehicle (e.g. change gears or apply brakes appropriately); Difficulties with multi-tasking (e.g. doing more than one thing at a time such as controlling the car and paying attention to the road/traffic); Difficulties when doing things in a series of steps (e.g. check mirrors, then signal, then change lanes); Difficulties interpreting traffic rules; Difficulties understanding crossroads; Difficulties understanding roundabouts; Feeling anxious; Difficulties staying focused or becoming easily distracted when driving; Difficulties judging distance or physical position of other road users in relation to your own vehicle; Difficulties interpreting the behaviour of other drivers or pedestrians on the road; Difficulties managing unexpected changes in the environment; Getting upset with people when they don't follow the rules; Difficulties judging when it is or isn’t safe to perform a maneuver; Difficulties making decisions e.g. about what speed is appropriate; Difficulties predicting what might be about to happen.

Respondents who indicated that they had started to learn but had stopped were asked to indicate their reason for stopping. They were presented with the same list of difficulties as given to current drivers and learners, but in this case they were asked simply to indicate which difficulties they had experienced (without stating the frequency). Two additional potential difficulties associated with the driving instructor were added: difficulty with understanding the instructor, and feeling uncomfortable with the driving instructor. A box for ‘other’ with space to type an open-ended answer was also included. Respondents who indicated they had never tried to learn to drive were asked whether they thought they would in the future. If they answered no to this question, they were again presented with the same list of difficulties and asked to indicate which (if any) contributed to their decision not to learn to drive, including a box for ‘other’.

The final section of the questionnaire was for completion by all participants (regardless of their driver status) and comprised the 50-item Autism Spectrum Quotient (AQ; Baron-Cohen et al., 2001), which requires participants to indicate whether or not a range of autistic traits apply to them. The questions come from five subscales with 10 items each, which describe different domains of autistic trait: social, attention switching, attention to detail, communication, and imagination.

## Results

The following analyses are based on comparisons between groups of respondents who reported either having or not having an autism diagnosis.

### Driver Status

A larger proportion of non-autistic participants (189; 88%) than autistic participants (106; 65%) reported currently holding a full driving license, χ^2^ = 27.42, *p* < 0.001. There were no differences in the proportion of autistic and non-autistic learner drivers (7 per group; 4% and 3% of total sample respectively). 49 (30% of total sample) autistic participants did not hold a license and were not currently learning to drive. Of these, 23 (14% of total sample) had started learning but discontinued prior to obtaining a license and 26 (16% of total sample) had not started learning. For the non-autistic group, 19 (6% of total sample) participants did not hold a license and were currently not learning to drive. Twelve (4% of total sample) of these had started learning but not obtained their license and 7 had not started (2% of total sample). Autistic participants were significantly more likely to have started to learn to drive but discontinued than participants who did not have an autism diagnosis, χ^2^ = 8.14, *p* = 0.004.

The next analyses were conducted independently for the groups of different driver status.

### Current Drivers

The autistic and non-autistic groups did not differ in the number of times they reported taking their driving test (both median = 1). The majority of individuals in both groups (~ 80%) indicated that they primarily learned to drive via a driving instructor. However, autistic drivers reported requiring more lessons with a driving instructor (median = 20–40) before passing their test than non-autistic drivers (median = 0–20), χ^2^ = 26.00, *p* < 0.001. The amount of additional driving practice participants had with friends or family before taking the test did not differ between the two groups (median = 10–20 h).

In relation to current driving habits, the groups did not differ in their estimated total monthly mileage or in the number of accidents they had had in the last year. However, fewer autistic drivers (74%) owned a car than non-autistic drivers (84%), χ^2^ = 4.78, *p* = 0.029.

#### Frequency of Driving Difficulties

The next analysis addressed the ratings of how often driving difficulties were experienced by autistic and non-autistic drivers. Mean scores on each question for the two groups are shown in Table 2, along with Cohen's *d* effect sizes for comparisons between groups. Autistic drivers reported experiencing difficulties on every measure more frequently than non-autistic drivers.

**Table 2:** Mean scores (standard deviations in brackets) on each driving difficulty item for autistic and non-autistic participants, and Cohen's *d* effect sizes for between groups comparisons

Difficulty	Autistic	Non-autistic	Effect size (Cohen's *d*)
Difficulties with physically operating the vehicle (e.g. change gears or apply brakes appropriately)	0.64 (0.89)	0.32 (0.58)	0.43**
Difficulties with multi-tasking (e.g. doing more than one thing at a time such as controlling the car and paying attention to the road/traffic)	1.47 (1.12)	0.81 (0.99)	0.62***
Difficulties when doing things in a series of steps (e.g. check mirrors, then signal, then change lanes)	0.76 (0.93)	0.38 (0.70)	0.46***
Difficulties interpreting traffic rules	0.92 (0.93)	0.58 (0.83)	0.39**
Difficulties understanding crossroads	0.83 (0.95)	0.51 (0.77)	0.37**
Difficulties understanding roundabouts	0.76 (0.97)	0.56 (0.76)	0.23
Feeling anxious	2.43 (1.31)	1.28 (1.21)	0.91***
Difficulties staying focused or becoming easily distracted when driving	1.50 (1.25)	0.80 (0.93)	0.64***
Difficulties judging distance or physical position of other road users in relation to your own vehicle	1.60 (1.22)	0.78 (0.96)	0.75***
Difficulties interpreting the behaviour of other drivers or pedestrians on the road	1.95 (1.10)	1.02 (0.93)	0.91***
Difficulties managing unexpected changes in the environment	1.78 (1.20)	0.81 (0.84)	0.94***
Getting upset with people when they don't follow the rules	2.67 (1.22)	1.95 (1.18)	0.60***
Difficulties judging when it is or isn’t safe to perform a manoeuvre	1.40 (1.09)	0.73 (0.80)	0.70***
Difficulties making decisions e.g. about what speed is appropriate	1.00 (0.97)	0.62 (0.78)	0.43***
Difficulties predicting what might be about to happen	1.45 (1.01)	0.76 (0.89)	0.72***

^*^ *p* < .05; ** *p* < .01; *** *p* < .001

A principal component analysis (PCA) was carried out on the responses to these questions in order to determine whether certain driving difficulties cluster together. Given the possibility that the factors might differ for the two groups, this was conducted separately for each group. PCA using oblique rotation (direct oblimin) was chosen, due to the possibility that factors may be correlated.

The Kaiser–Meyer–Olkin measure revealed adequate sampling accuracy, KMO = 0.86 for the autistic group; KMO = 0.92 for the non-autistic group. Bartlett’s test of sphericity indicated that correlations between the items were sufficient for both the autistic group, χ^2^ (105) = 708.99, *p* < 0.001 and the non-autistic group, χ^2^ (105) = 1488.98, *p* < 0.001.

For the autistic group, three factors emerged from the PCA, which accounted for a total of 61.33% of the variance. The list of component loadings with values of greater than 0.4 are shown in Table 3. The first component contains items relating to attention, distraction, and multitasking and hence we named it “Driving Executive”. The second component contains items primarily to do with making sense of or judging types of situation and hence we named it “Driving Understanding”. The third factor was mainly items that related to interactions with other road users and hence was named “Driving Social Interaction”.

**Table 3:** Factor structure of driving difficulties for autistic drivers

Item	Factor 1	Factor 2	Factor 3
Difficulties staying focused or becoming easily distracted when driving	0.86		
Difficulties with multi-tasking (e.g. doing more than one thing at a time such as controlling the car and paying attention to the road/traffic)	0.84		
Feeling anxious	0.70		
Difficulties when doing things in a series of steps (e.g. check mirrors, then signal, then change lanes)	0.66		
Difficulties with physically operating the vehicle (e.g. change gears or apply brakes appropriately)	0.64		
Difficulties managing unexpected changes in the environment	0.58		
Difficulties making decisions e.g. about what speed is appropriate	0.43		
Difficulties understanding crossroads		0.88	
Difficulties understanding roundabouts		0.88	
Difficulties interpreting traffic rules		0.87	
Difficulties judging distance or physical position of other road users in relation to your own vehicle		0.45	
Difficulties predicting what might be about to happen		0.41	
Getting upset with people when they don't follow the rules			0.84
Difficulties interpreting the behaviour of other drivers or pedestrians on the road			0.56
Difficulties when doing things in a series of steps (e.g. check mirrors, then signal, then change lanes)			− 0.42
Eigenvalues	6.41	1.45	1.34
Variance (%)	42.78	9.64	8.92

For the non-autistic group, only one factor emerged from the PCA, which accounted for a total of 51.21% of the variance. A second component appeared but only one item loaded onto this component; hence it was not retained in the solution. The list of component loadings with values of greater than 0.4 are shown in Table 4. As the component contained almost all of the items from the questionnaire, it was named “Driving General”.

**Table 4:** Factor structure of driving difficulties for non-autistic drivers

Item	Factor 1
Difficulties managing unexpected changes in the environment	0.82
Difficulties when doing things in a series of steps (e.g. check mirrors, then signal, then change lanes)	0.80
Difficulties understanding crossroads	0.78
Difficulties interpreting traffic rules	0.78
Difficulties judging when it is or isn’t safe to perform a manoeuvre	0.78
Difficulties judging distance or physical position of other road users in relation to your own vehicle	0.77
Difficulties with multi-tasking (e.g. doing more than one thing at a time such as controlling the car and paying attention to the road/traffic)	0.75
Difficulties predicting what might be about to happen	0.75
Difficulties interpreting the behaviour of other drivers or pedestrians on the road	0.74
Difficulties making decisions e.g. about what speed is appropriate	0.71
Difficulties with physically operating the vehicle (e.g. change gears or apply brakes appropriately)	0.70
Feeling anxious	0.68
Difficulties understanding roundabouts	0.65
Difficulties staying focused or becoming easily distracted when driving	0.53
Eigenvalues	7.68
Variance (%)	51.21

#### Relationship Between Driving Difficulties and Autistic Traits

The next analyses aimed to determine whether the various factors of driving difficulty related to types of autistic traits. For the group who had a diagnosis of autism, the analysis was aimed at determining whether specific autistic features related to specific difficulties with driving. For the non-autistic group, the aim was to determine whether having a larger number of autistic traits (in the absence of any diagnosis) related to a greater degree of difficulty with driving. Autistic traits were measured using the Autism Spectrum Quotient (Baron-Cohen et al., 2001). Scores were calculated for each of the five 10-item subscales of the AQ. Mean scores for each of the subscales for autistic and non-autistic drivers are displayed in Table 5.

**Table 5:** Mean scores (standard deviation in brackets) on AQ subscales for autistic and non-autistic drivers

	Social	Attention switching	Attention to detail	Communication	Imagination
Autistic	8.83 (1.21)	9.08 (1.38)	7.80 (1.63)	8.22 (1.65)	6.48 (2.17)
Non-autistic	3.51 (2.77)	5.00 (2.42)	5.25 (2.29)	3.40 (2.43)	3.39 (2.11)

Multiple linear regression analyses were conducted for each of the three factors of driving difficulty for autistic participants with the five AQ subscales as predictors (using the forced entry method). For the Driving Executive factor, the model approached significance, *F*(5,80) = 2.32, *p* = 0.051, indicating that the predictors accounted for approximately 13% of the variance in the Driving Executive factor. Only scores on the Attention Switching subscale of the AQ were a significant predictor of the Driving Executive factor (see Table 6).

**Table 6:** Predictors of driving executive factor

	β	t	p
Constant		1.35	.180
Social	− .15	1.28	.203
Attention switching	.34	2.68	.009**
Attention to detail	− .03	− .031	.758
Communication	.11	0.90	.373
Imagination	− .06	− 0.47	.642

^*^ *p* < .05; ** *p* < .01; *** *p* < .001

For the Driving Understanding factor, the model was significant, *F*(5,80) = 2.62, *p* = 0.03, indicating that the predictors accounted for approximately 14% of the variance in the Driving Understanding factor. Only scores on the Communication subscale of the AQ were a significant predictor of the Driving Understanding factor (see Table 7).

**Table 7:** Predictors of driving understanding factor

	β	t	p
Constant		1.61	.111
Social	− .13	1.15	.252
Attention switching	.04	0.28	.778
Attention to detail	.12	1.05	.296
Communication	.28	2.35	.021*
Imagination	.13	1.05	.298

^*^ *p* < .05; ** *p* < .01; *** *p* < .001

For the Driving Social Interaction factor, the regression model approached significance, *F*(5,80) = 2.26, *p* = 0.057, where the predictors accounted for approximately 12% of the variance in the Driving Social Interaction factor. Only scores on the Attention Switching subscale of the AQ significantly predicted the Driving Social Interaction factor (see Table 8).

**Table 8:** Predictors of Driving Social Interaction factor

	β	t	p
Constant		2.75	.007
Social	.12	1.07	.289
Attention switching	.29	2.30	.024*
Attention to detail	.07	0.65	.521
Communication	.09	0.73	.469
Imagination	− .24	1.89	.062

^*^ *p* < .05; ** *p* < .01; *** *p* < .001

For the non-autistic participants a multiple regression analysis was also carried out for the Driving General factor with the five AQ subscales as predictors. The regression model was significant, *F*(5,134) = 10.20, *p* < 0.001 with the predictors accounting for approximately 28% of the variance in the Driving General factor. Only scores on the Communication subscale were a significant predictor for the Driving General factor (see Table 9).

**Table 9:** Predictors of Driving General factor

	β	t	*p*
Constant		2.85	.005
Social	.11	1.02	.310
Attention switching	.13	1.34	.183
Attention to detail	− .08	1.06	.289
Communication	.344	3.17	.002*
Imagination	.021	0.24	.813

^*^ *p* < .05; ** *p* < .01; *** *p* < .001

### Non-Drivers

Owing to the small number of learner drivers in the sample, analyses were not carried out on their responses. However, separate analysis was carried out on responses from those individuals who indicated that they did not have a driving license and were not currently learning (autistic *N* = 49, non-autistic *N* = 19). The majority of individuals in both groups who were not currently learning to drive indicated that they had at some point attempted to learn but had not obtained a license.

When asked about the specific difficulties they had in learning to drive which had led them to discontinue, autistic participants reported a greater number of difficulties (*M* = 7.81, *SD* = 3.74) than non-autistic participants, (*M* = 3.93, *SD* = 3.97), t(43) = 3.16, *p* = . 003. Specifically, autistic individuals reported they had had problems with multitasking, difficulty understanding the rules, feeling anxious, difficulty judging the positions of other vehicles, more difficulty interpreting other road users’ behaviour, and difficulty managing unexpected changes in the environment.

#### Relationship of Driving Difficulties with Autistic Traits

Table 10 shows the mean scores on the five AQ subscales for autistic and non-autistic non-drivers. Correlations were carried out to determine whether the total number of driving difficulties related to scores on the AQ subscales for participants in each group. For autistic participants, there were significant positive correlations between total driving difficulties and scores on the social (*r* = 0.40, *p* = 0.032) attention switching (*r* = 0.54, *p* = 0.003), and communication (*r* = 0.43, *p* = 0.019) subscales of the AQ. Total driving difficulties did not correlate with attention to detail or imagination subscales of the AQ in autistic non-drivers. For non-autistic non-drivers, total driving difficulties correlated positively with scores on the social (*r* = 0.81, *p* = 0.004) and communication (*r* = 0.69, *p* = 0.028) subscales of the AQ.

**Table 10:** Mean scores (standard deviation in brackets) on AQ subscales for autistic and non− autistic non-drivers

	Social	Attention switching	Attention to detail	Communication	Imagination
Autistic	8.08 (1.75)	8.60 (1.61)	6.46 (2.20)	7.65 (1.91)	6.15 (2.43)
Non-autistic	5.36 (3.41)	6.67 (3.00)	4.71 (3.12)	4.28 (3.29)	2.51 (2.11)

Only 11 respondents (9 autistic) indicated that they had not started to learn to drive and did not think that they would learn in the future. Autistic individuals tended to indicate they believed that they would experience many of the difficulties listed i.e. multitasking, physically operating the car etc. (*M* = 6.50, *SD* = 3.74) while the two non-autistic individuals did not indicate they thought they would have difficulties in the areas listed (*M* = 1.00, *SD* = 1.41). Instead they cited logistical reasons for not driving such as having a motorcycle license or using public transport instead.

## Discussion

A higher proportion of non-autistic than autistic individuals who responded to the survey currently held a license, while a greater proportion of autistic individuals had started to learn but discontinued. These results are consistent with previous studies that have found that fewer autistic individuals hold a driving license (e.g. Curry et al., 2018) and emphasise the need for further research to understand why this is the case. As the largest groups of respondents within the survey were current drivers, the majority of analyses focused on these groups. The autistic drivers reported having taken more lessons before passing the driving test than non-autistic drivers, although there were no reported differences in the number of times they had taken the driving test. This is consistent with previous research that found that autistic individuals on average pass their test later than non-autistic individuals (Daly et al., 2014). This may be because autistic people face more challenges while learning to drive (Cox et al., 2012), but could also reflect autistic learners having decreased confidence in their driving and preferring to undertake more lessons prior to being tested.

The autistic drivers reported experiencing more difficulties in every aspect of driving that was addressed in the questionnaire. This finding echoes those of Daly et al. (2014) who found that autistic people self-reported engaging more frequently in a wide range of problematic driving behaviours, including mistakes, slips/lapses and intentional violations. In their study, Daly et al. argued that this might be consistent with global neurocognitive and social challenges in driving behaviours for autistic individuals, as opposed to a limited set of specific skills. In contrast, the results of the factor analysis carried out in the current research suggests that driving difficulty in autism might be best characterised as having several distinct (although correlated) dimensions.

The emergence of three factors from the analysis of driving difficulties is also evidence against the possibility that autistic individuals believe themselves to be universally poor drivers and consequently respond indicating that they experience all of the driving difficulties listed. The factor structure instead implies that certain types of difficulty cluster together in autistic drivers and is consistent with the possibility that different autistic people are likely to have problems in differing particular domains. For instance, one person might find they have difficulties with executive aspects of driving such as keeping their attention focused on relevant parts of the environment or doing multiple activities at once, while another individual may perceive their main problem to be their interactions with other road users.

Moreover, each of the three driving difficulty factors was predicted by scores on specific AQ subscales. The “Driving Executive” factor was predicted by scores on the ‘Attention Switching’ subscale of the AQ. This is the subscale that focuses most closely on behaviours that are thought to be associated with executive dysfunction, including items such as “I find it easy to do more than one thing at once”, “If there is an interruption I can switch back to what I was doing very quickly” and “It does not upset me if my daily routine is observed”. Therefore, it makes sense that those autistic drivers who report having the highest number of autistic traits relating to attention switching are also the ones who report having the most difficulties in relation to executive aspects of driving, which included items such as “Difficulties with physically operating the vehicle (e.g. change gears or apply brakes appropriately)”, “Difficulties staying focused or becoming easily distracted when driving” and “Difficulties when doing things in a series of steps (e.g. check mirrors, then signal, then change lanes)”.

The “Driving Understanding” factor was predicted by scores on the ‘Communication’ subscale of the AQ. The ‘Driving Understanding’ factor included several items that related to drivers’ ability to interpret or make inferences about specific driving situations, such as “Difficulties understanding crossroads”, “Difficulties interpreting traffic rules”, and “Difficulties predicting what might be about to happen”. The ‘Communication’ subscale of the AQ has several items based around understanding of social situations, such as ‘I find it easy to ‘read between the lines’ when someone is talking to me’, ‘When I talk on the phone I’m not sure when it’s my turn to speak’ and ‘other people frequently tell me that what I’ve said is impolite, even though I think it is polite’. Perhaps there are some commonalities between understanding the rules of interaction on the road and the rules of interaction in social situations, which might account for the relationship between the two.

The final driving factor for autistic respondents was named “Driving Social Interaction” and was predicted by scores on the ‘Attention Switching’ subscale of the AQ. Only three items contributed to the ‘Driving Social Interaction’ factor and the item that contributed most strongly was ‘Getting upset with people when they don't follow the rules’. Although this item appears to be about interaction with other road users, endorsing this item might actually reflect levels of rigidity or difficulty regulating emotions which relates to executive functioning. The ‘Attention Switching’ subscale includes items such as ‘It does not upset me if my daily routine is disturbed’ and ‘I tend to have very strong interests which I get upset about if I can’t pursue’. These items share the common theme of feeling upset or bothered when a situation deviates from what is expected or preferred. Hence, this might explain why the ‘Driving Social Interaction’ factor scores were primarily determined by scores on the ‘Attention Switching’ subscale of the AQ.

For the non-autistic drivers, a different factor structure of driving difficulties emerged where most of the items loaded onto a single factor, which was named ‘Driving General’. This indicates that for non-autistic drivers, where people have difficulties, they tend to see them as being quite widespread across domains rather than occurring in more independent clusters. It could also be that this factor reflects driving confidence rather than actual ability/difficulty in these areas. Total AQ scores and in particular scores on the ‘Communication’ subscale predicted scores on the ‘Driving General’ factor in non-autistic participants. This finding is important in that it suggests that potentially even people with subclinical levels of autistic traits may find driving substantially more challenging than those with lower levels of autistic traits (or at the very least, believe that they do).

The study reported in this paper is the first to our knowledge that has asked autistic adult non-drivers about their reasons for not driving. The majority of non-drivers who responded (both autistic and non-autistic) had previously started to learn but had discontinued without obtaining a license. Mirroring the responses of the driver groups, the autistic non-drivers reported having had higher levels of difficulty in the aspects of driving they were surveyed on than the non-autistic non-drivers. Thus, even among individuals who have not obtained a driving license, those who were autistic believed they had more difficulties with a wide range of aspects of driving. In both groups, higher levels of autistic traits—and more specifically social and communication difficulties (with the addition of attention switching for the autistic group)—related to reporting a greater degree of driving difficulty.

Finally, we consider implications and limitations of the research. Although the survey was open to anyone regardless of their driver status, the sample mainly consisted of people who were current drivers. While this might reflect the characteristics of the population as a whole, it may be that drivers were more motivated to respond to the survey than those who do not currently drive, so the proportions observed in this study may not accurately represent the proportions of individuals within the population who drive. However, there is no particular reason to suspect that this can explain group differences in driver status, which are also consistent with previous studies. Nevertheless, given that non-driver groups were relatively modest in size, a priority for future research would be to obtain larger samples of non-drivers to understand more about why these individuals have not obtained a license.

Responses to the online survey came from a variety of countries globally. While this was done in order to ensure a broad range of views were obtained and to gain a sufficient sample size for our analyses, it is worth noting that some road rules and driving behaviours may differ by country (e.g. Lim et al., 2013), and autism itself may present differently across cultures. Nevertheless, it is hard to see how this sampling method could account for the relationships between traits and driving difficulties seen in the data.

The results are in line with the notion that there are at least three different domains of driving difficulty for autistic people (which we have labelled as Executive, Understanding, and Social interaction), and two of these appear to map onto the two key areas within DSM-5 (APA, 2013) used to identify autism. The findings provide a novel demonstration of how clinical features of autism (as measured a standardised tool) can directly relate to outcomes in an applied real-world domain. They also suggest that a one-size-fits-all approach to driver training is unlikely to be most effective for autistic people, and instead, consideration of the particular profile of features that an autistic person has could help tailor driving tuition to that person’s abilities. Previous research has focused on training executive functioning, finding some evidence of enhanced driving performance in autistic drivers following such training (Cox et al., 2017). The current research suggests that training that targets the social aspects of driving as well as understanding of specific kinds of road situations might also benefit some autistic people.

It is not possible to know from the current research whether autistic people and those high in autistic traits actually do have more difficulties when driving or whether they just perceive that they do. Studies that have attempted to measure actual driving ability in a simulator have reported autistic drivers perform more poorly than non-autistic drivers on some measures (e.g. Cox et al., 2016); however on-road observations have yielded little difference between groups in performance (Chee et al., 2017). One recent study (Curry et al., 2021) even reported that newly qualified autistic drivers have slightly lower overall crash risk than non-autistic drivers, but the two groups differed in the kinds of crash they were involved in. This is consistent with the suggestion arising from the current study that the way driving difficulties manifest may be different for autistic and non-autistic drivers. Moreover, recent research suggests that measures of autistic features—the ADOS (Lord, Rutter, DiLavore, Risi, Gotham, & Bishop, 2012) and SRS (Constantino, 2012)—do not predict actual driving performance (Patrick et al., 2020) in autistic drivers, potentially implying that autistic traits may impact beliefs more than reality. It is also not possible to know which group is more accurate in their appraisal of their own driving ability, although it has been widely reported previously that typically developing drivers tend to overestimate their own driving skill (Delhomme, 1991), and so may also engage in socially desirable responding in this context.

However, even if autistic people merely report having more difficulties, the importance of perceptions should not be underestimated. Confidence in ones driving ability has been found to be a key factor in driving cessation in older adults (Charlton et al., 2003). If a person believes they find driving difficult, they may find it more stressful, and might be more likely to avoid driving or discontinue before or even after obtaining a license. This suggestion is consistent with the finding that more autistic people than non-autistic people in this study had started to learn but had stopped before obtaining a license. Therefore, perceptions of difficulty may impact outcomes as much as actual difficulty. However, future research could usefully examine the relationship between autistic traits, perceptions of driving difficulty, and direct measures of driving skill in both autistic and non-autistic individuals.

## Footnotes

1 Identity-first language is used throughout this manuscript as it has been reported to be preferred by the majority of the autistic community; Kenny et al. (2016)
